# A balancing act: how plants integrate nitrogen and water signals

**DOI:** 10.1093/jxb/eraa054

**Published:** 2020-01-28

**Authors:** Viviana Araus, Joseph Swift, Jose M Alvarez, Amelia Henry, Gloria M Coruzzi

**Affiliations:** 1 Center for Genomics and Systems Biology, Department of Biology, New York University, NY, USA; 2 Centro de Genómica y Bioinformática, Facultad de Ciencias, Universidad Mayor, Santiago, Chile; 3 International Rice Research Institute, Metro Manila, Philippines; 4 Nanjing Agricultural University, China

**Keywords:** Agronomy, climate change, drought, nitrogen, signaling, systems biology

## Abstract

Nitrogen (N) and water (W) are crucial inputs for plant survival as well as costly resources for agriculture. Given their importance, the molecular mechanisms that plants rely on to signal changes in either N or W status have been under intense scrutiny. However, how plants sense and respond to the combination of N and W signals at the molecular level has received scant attention. The purpose of this review is to shed light on what is currently known about how plant responses to N are impacted by W status. We review classic studies which detail how N and W combinations have both synergistic and antagonistic effects on key plant traits, such as root architecture and stomatal aperture. Recent molecular studies of N and W interactions show that mutations in genes involved in N metabolism affect drought responses, and vice versa. Specifically, perturbing key N signaling genes may lead to changes in drought-responsive gene expression programs, which is supported by a meta-analysis we conduct on available transcriptomic data. Additionally, we cite studies that show how combinatorial transcriptional responses to N and W status might drive crop phenotypes. Through these insights, we suggest research strategies that could help to develop crops adapted to marginal soils depleted in both N and W, an important task in the face of climate change.

## Teaser

We discuss how both nitrogen and water availability combine to impact plant biological responses at the molecular, transcriptomic, and physiological level.

## Introduction

Plants source both nitrogen (N) and water (W) from soils for growth and development. Both play fundamental roles in plant biology—N is found in almost every biomolecule plants create, while W serves as the solvent and milieu for all biological processes.

Since both N and W are increasingly limited in soils world-wide, N-based fertilizers and irrigation underlie modern agriculture’s goal to meet yield potential. In the coming decades, climate change will force farmers around the globe to adapt to drier, nutrient-poor soils (Robertson and Vitousek, 2009; Ulrich *et al.*, 2014). At the same time, the damaging environmental impacts of synthesizing N fertilizers—including disruption of the global N cycle—are already being felt (Robertson and Vitousek, 2009). Consequently, in an attempt to develop crops that are either N- or W-use efficient, research efforts have focused on understanding how the availability of N or W in soils impacts plant biology—at both the physiological and molecular level.

**Fig. 1.**
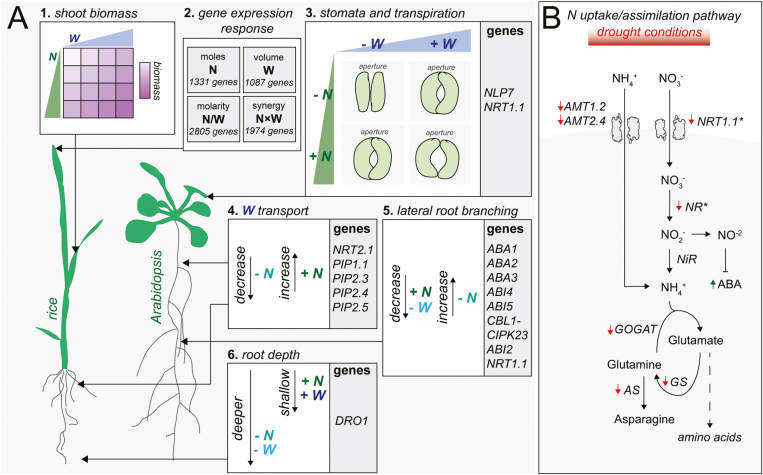
N and W doses combine to impact plant physiology and gene regulation. (A) (A.1) N and W combine to have a synergistic interaction on shoot biomass in rice (Swift *et al.*, 2019). (A.2) A single integrated study of N and W dose responses performed in rice reveals plant responses to N dose as (moles) or N concentration (N/W), which exhibit distinct transcriptomic responses. Additionally, the dose of N and W also exhibits a synergistic effect (N×W) on gene expression responses and phenotypes (Swift *et al.*, 2019). (A.3) Stomatal opening and transpiration are influenced by N and W availability (Shi *et al.*, 2014). *NLP7* and *NRT1.1/NPF6.3* are genes that mediate the role that N plays in stomatal aperture in Arabidopsis (Guo *et al.*, 2003; Castaings *et al.*, 2009). (A.4) Hydraulic conductivity is modulated by soil N and the N transporter NRT2.1, and through regulation of expression of PIP aquaporins in Arabidopsis and rice (Li *et al.*, 2016). (A.5) Drought and high N have a negative effect on root branching in Arabidopsis (Signora *et al.*, 2001; De Smet *et al.*, 2003). Genes involved in ABA biosynthesis and sensing can mediate the repressive effect of N on root branching in Arabidopsis (Signora *et al.*, 2001). Additionally, the CBL1–CIPK23–NRT1.1/NPF6.3–ABI2 regulatory module integrates N and W levels to regulate lateral root branching in Arabidopsis (Léran *et al.*, 2015). (A.6) Root systems growing deeper to forage for W also assist with N uptake under low W and low N conditions (Lilley and Kirkegaard, 2011). DRO1 governs rice cell elongation, leading to expansive roots that promote N and W uptake in rice (Arai-Sanoh *et al.*, 2014). (B) Drought conditions negatively impact N uptake and N metabolism gene expression in Arabidopsis (red arrows) (Goel and Singh, 2015; Duan *et al.*, 2016). Mutating genes (indicated by *) in N metabolism improves drought tolerance in Arabidopsis (Guo *et al.*, 2003). NR activity contributes to NO levels that inhibit ABA activity in Arabidopsis (Lozano-Juste and León, 2010; Castillo *et al.*, 2015).

However, many important plant traits are simultaneously dependent on both N and W input levels. Indeed, given their central role in plant physiology, N and W have many combinatorial effects on plant phenotypes. Arguably the most important interaction is their combined effect on biomass and crop yield potential—which is only achieved when both N and W are non-limiting (Patterson *et al.*, 1997; Di Paolo and Rinaldi, 2008; Brueck and Senbayram, 2009; Shi *et al.*, 2014; Swift *et al.*, 2019) (Fig. 1A). In recent years, the molecular and signaling components that underlie plant responses to either N or W have begun to be elucidated (O’Brien *et al.*, 2016; Vishwakarma *et al.*, 2017); however, how they overlap with one another remains poorly understood. Since combinations of N and W have a clear impact on important plant traits, it is likely that crosstalk exists between the molecular mechanisms that sense and respond to N and W.

To address this knowledge gap, this review is focused on the interactive effects N and W can have on plant biology. We start by assessing how W affects N availability in soils, and review the combinatorial effects that N and W doses can have on key plant traits, such as root system architecture and stomatal aperture. We then examine molecular evidence, largely from Arabidopsis and rice, which indicates how N and W sensing and signaling responses interact to mediate these physiological responses. Additionally, taking advantage of existing genomic data sets, we perform a meta-analysis to determine how N and W signals converge. Using these insights, we then propose research strategies to improve plant growth on arid, nutrient-poor marginal soils.

## W informs N availability in soils

In most indigenous soils, N is present in organic forms at low levels (<1% of the total soil volume); thus, agricultural systems typically require a high supply of mineral N fertilizer to meet yield potential—where the predominant forms of applied N are ammonium and nitrate (Raun and Johnson, 1999). However, the fate of applied N depends on a variety of edaphic, climatic, and agronomic factors; and the level of W in the soil plays an essential role.

In general, plants grown in flooded-anaerobic conditions, including paddy rice, use ammonium as the primary source of N (Sasakawa and Yamamoto, 1978). In aerobic soils where nitrification can occur, nitrate is the predominant form of available N for plants, including Arabidopsis (Crawford and Glass, 1998). Importantly, soil water regimes affect the availability of N forms. For example, in flooded fields, the formation of a hardpan layer restricts downward W flow, thereby helping to maintain soil saturation and reduce the loss of N nutrients by leaching (Buresh *et al.*, 2008). In contrast, in non-flooded soils, high moisture can contribute to decreased N availability by leaching (Fuentes *et al.*, 2003). Especially since nitrate holds a similar negative charge to soil particles, it will leach as W runs off from soil along with W run-off (Gärdenäs *et al.*, 2005). For this reason, drier soils have reduced nitrate leaching.

Thus, field management is essential for optimizing N resources. For example, the timing of irrigation or rainfall with respect to N fertilizer application has a strong influence on the fate of N availability in the soil. If fertilized fields experience soil drying, N concentrations in the soil solution will increase, ultimately increasing volatilization of ammonia from the soil surface. Conversely, if solid N fertilizer pellets are applied to dry soil, less N fertilizer is dissolved. Subsequent watering can reduce volatilization of N, since N is washed down below the surface, in effect reducing surface N concentrations (Cameron *et al.*, 2013). Given this, scheduling N fertilizer application with both plant N requirements and W availability can improve profitability for farmers (Banayo *et al.*, 2018). Indeed, such decisions are essential for more sustainable and smart use of N and W application for improving the fate of crop productivity.

## A virtuous circle: foraging for W can help N uptake

Plant roots regulate resource uptake from soil. Indeed, root system architecture can adapt to N and W availability; individual root traits can adapt to allow plants to better forage for either N or W. However, as described above, N distribution in soils is affected by W and, indeed, N must be dissolved in W for uptake to occur. For this reason, root system traits that promote or limit the uptake of N can impact the uptake of W, and vice versa. Indeed, root traits can be affected synergistically or antagonistically by the combination of N and W, as described below.

Many root architecture traits that promote the uptake of W often also improve N uptake (Fig. 1A). One example of this synergistic interaction occurs during drought. As drought occurs, W will move deeper into soils, carrying nitrate with it. Roots show positive hydrotropism—following a moisture gradient downwards to reach W in deeper soil layers (Henry *et al.*, 2011; Uga *et al.*, 2015). Root systems that grow deeper to forage for W can assist with nitrate uptake (Pedersen *et al.*, 2010; Lilley and Kirkegaard, 2011). This effect is illustrated by the *DEEPER ROOTING 1* (*DRO1*) locus, which controls root angle in rice (Arai-Sanoh *et al.*, 2014). DRO1 governs cell elongation in the root tip, directing asymmetric root growth and downward bending, resulting in improved W uptake. DRO1 lines also showed higher N uptake in some instances, resulting in higher grain yield (Arai-Sanoh *et al.*, 2014) (Fig. 1A). In line with these findings, other root traits that improve W uptake can also improve N absorption. For example, to simultaneously enhance N and W absorption, plants can increase root length density—the length of all roots per volume soil (Tsuji *et al.*, 2005; Bonifas and Lindquist, 2009; Wasaya *et al.*, 2018). The same holds true for root diameter. Finer roots enable plants to increase hydraulic conductance—the ability of roots to conduct W across the root surface and into tissue—and decrease the apoplastic barrier of W entering the xylem (Henry *et al.*, 2012; Comas *et al.*, 2013; Li *et al.*, 2019), while at the same time enhancing N uptake (Bonifas and Lindquist, 2009; Li *et al.*, 2019).

Likewise, an increase in N availability in soils can promote W uptake. Specifically, evidence shows that the movement of W into root tissues is positively influenced by soil N availability. Under well-watered conditions, a variation in nitrate concentration at the root surface alters root hydraulic conductivity (Gloser *et al.*, 2007). This results in W being preferentially absorbed by plant roots located in nitrate-rich zones (Gorska *et al.*, 2008). By these means, high N soils can increase W uptake in irrigated soils (Gorska *et al.*, 2008; Ren *et al.*, 2015). Importantly, this effect appears to be specific to N; other macronutrients, such as sulfate or phosphate, do not have this same effect on W uptake (Gorska *et al.*, 2008). To achieve this, N may cause changes in cell membrane hydraulic properties, which directly affect the intracellular nitrate concentrations, attracting more W into the cellular space (Clarkson *et al.*, 2000; Gloser *et al.*, 2007; Guo *et al.*, 2007; Li *et al.*, 2016).

Another explanation for N’s effect on W uptake is through the impact of N on gene expression of aquaporins (Fig. 1A). Aquaporins are W transporters that meter root hydraulic conductance. Residing in both plasma membranes and tonoplasts, aquaporins regulate osmotic potential by facilitating the transport of W across membranes (Maurel, 1997). Aquaporins in many different plant species are differentially expressed in response to N availability. N deprivation decreases the expression of root-specific aquaporin genes, whereas N resupply increases their expression (Ishikawa-Sakurai *et al.*, 2014; Li *et al.*, 2016). For example, the expression of root-specific rice aquaporin genes *OsPIP1.1*, *OsPIP2.3-2.5*, *OsTIP1.1-1.2*, and *OsTIP2.2* is positively associated with N availability (Ishikawa-Sakurai *et al.*, 2014). In contrast, N starvation leads to a reduction in aquaporin gene expression levels, weakening root hydraulic conductivity (Ishikawa-Sakurai *et al.*, 2014). In agreement with this, disrupting the NRT2.1 nitrate transporter negatively impacts the transcript abundance of PIP1.1, PIP1.2, PIP2.1, PIP2.3, and PIP2.7 aquaporins, resulting in a reduction in root hydraulic conductivity (Li *et al.*, 2016). In addition, proteomic analyses has revealed that N availability also influences the levels of PIP aquaporin proteins and their phosphorylation status, with a concomitant effect on root hydraulic conductivity (di Pietro *et al.*, 2013). Collectively, these results indicate that N regulation of aquaporin at different levels impacts hydraulic conductance (Fig. 1A).

## High N phenocopies the effect of low W on root branching

Lateral root growth can become repressed when either too much N or too little W is present. In this context, cumulative evidence suggests that the molecular mechanisms that limit root branching under W stress are similar to those that limit lateral root growth under high N. Physiologically, the reason why root branching is limited under these two conditions differs. In the case of W, lateral root branching can be inhibited within parts of the rhizosphere where W is absent (Babé *et al.*, 2012), as primary roots forage deeper into soils for W (Henry *et al.*, 2012). Conversely, under high N conditions, lateral root development is suppressed when plants have met their N demand, relying on systemic N signaling to avoid absorbing surplus N they cannot assimilate (Signora *et al.*, 2001) (Fig. 1A). It appears that the phenocopying effect of low W and high N on root branching is driven in part by the same plant hormone—abscisic acid (ABA), as reviewed below.

At the signaling level, inhibition of lateral roots is achieved by ABA, which is synthesized in roots in response to drought stress (De Smet *et al.*, 2003). ABA may also be responsible for inhibiting lateral root branching in response to high levels of N (Signora *et al.*, 2001; Sun *et al.*, 2017). This response is dependent on genes responsible for ABA synthesis and sensing, namely *ABA1*, *ABA2*, *ABA3*, *ABI4*, and *ABI5* (Signora *et al.*, 2001; Seo and Koshiba, 2002) (Fig. 1A). In line with this, ABA biosynthesis and sensing mutants display resistance to the inhibitory effects high N can have on lateral root initiation, and ABA concentration at the root tip positively correlates with high N exposure. Additionally, ABA has been shown to accumulate in the root endodermis in response to high N treatments (Ondzighi-Assoume *et al.*, 2016), suggesting that high N may inhibit lateral root initiation from the pericycle via increasing ABA.

An increase in ABA in response to high N may be because N can serve as an osmolyte, and thus can itself cause osmotic stress. Indeed, repression of lateral root initiation due to very high N resembles the root phenotypes observed when plants are exposed to other osmolytes, such as potassium chloride or mannitol (Deak and Malamy, 2005). However, another possible explanation exists at the molecular signaling level, where ABA directly inhibits nitrate sensing and transport. For instance, the nitrate transceptor NRT1.1/NPF6.3 is under the regulation of ABA signaling. ABA INSENSITIVE 2 (ABI2), a phosphatase that is inhibited by ABA, indirectly regulates NRT1.1/NPF6.3. In this signaling cascade, ABI2 dephosphorylates CBL1–CIPK23, which is responsible for phosphorylation and inhibition of NRT1.1/NPF6.3 (Ho *et al.*, 2009; Léran *et al.*, 2015). Thus, stress-induced synthesis of ABA inactivates ABI2, causing phosphorylation of NRT1.1/NPF6.3, and leading to a reduction in nitrate uptake. Supporting this, *abi2* mutants have a similar phenotype to *nrt1.1/npf6.3* mutants, which fail to induce lateral root elongation in high nitrate (Léran *et al.*, 2015). Since ABA binding to ABA receptors inactivates ABI2, this could be a mechanism to decrease nitrate uptake and lateral root growth under stress conditions. Thus, N and ABA may be convergent signals that coordinate root foraging and optimize the use of plant resources.

## N encourages higher transpiration rates under drought conditions

Whilst root systems perceive and acquire N and W from the soil, shoot systems assimilate N and transpire W. Thus, both N and W levels in shoots coordinately control stomata function, gas interchange, and shoot growth rate (Fig. 1A).

The opening and closing of stomata regulates the amount of W released from leaf tissue. Stomatal aperture and transpiration rates are positively associated with the amount of W available. N availability also impacts stomatal aperture, where an increase in soil N can lead to higher transpiration rates in leaves (Corak *et al.*, 1991; Hatfield *et al.*, 2001; Ren *et al.*, 2015) (Fig. 1A). This is not only because soil N stimulates more W uptake, as discussed above, but also because an increase in N can allow for higher rates of photosynthesis and carbon fixation (Wright *et al.*, 2003; Ding *et al.*, 2018). Since leaves holding a higher amount of unassimilated N demand more CO_2_ from fixation for assimilation into organic N, stomata remain open (Patterson *et al.*, 1997; Guo *et al.*, 2007).

While stomata remain closed under low N and drought conditions, combined drought and high N conditions can encourage stomata to remain open, leading to higher transpiration rates (Shi *et al.*, 2014) (Fig. 1A). This response may explain why N fertilization is reported to improve plant growth under drought conditions. High N can result in stomata remaining open, leading to higher transpiration rates and encouraging greater W use (Shi *et al.*, 2014) (Fig. 1A). In this way, in the short term, high N conditions can delay the effects of drought by allowing plants to continue to grow (Shangguan *et al.*, 2000; Cabrera-Bosquet *et al.*, 2007). However, these benefits may be short lived. High N under drought conditions may be problematic in the long term, because N may delay a plant’s response to W scarcity. N will encourage additional shoot growth, which can exacerbate W stress due by increasing transpiration area (Cabrera-Bosquet *et al.*, 2007). High N can also repress root elongation (Mi *et al.*, 2008), limiting foraging for W. In this way, high N prevents plants from developing stress avoidance mechanisms to cope with long-term drought stress (Claeys and Inzé, 2013). This may explain why some reports show that N fertilization can lead to crops performing less well under drought conditions (Patterson *et al.*, 1997; Gong *et al.*, 2011).

## Decreasing N metabolism improves drought tolerance

In recent years, several studies have indicated that genes involved in N metabolism are also involved in plant responses to drought. Evidence indicates that when plants encounter drought stress, they attempt to reduce the amount of N they absorb and assimilate. Not only are N uptake/metabolism genes down-regulated under drought (Goel and Singh, 2015), remarkably, disrupting their function has led plants to display improved drought tolerance phenotypes (Fig. 1B).

Under drought stress, many genes responsible for N transport and assimilation are repressed at the transcriptional level (Fig. 1B). This includes N transporters (AMT1.2, AMT2.4, and NRT1.5), as well as genes encoding enzymes that assimilate ammonia into the amino acids glutamine, glutamate, and asparagine (GOGAT, GS, and AS) (Nagy *et al.*, 2013; Singh and Ghosh, 2013; Duan *et al.*, 2016) (Fig. 1B). Recent evidence suggests that the drought stress hormone ABA may indirectly cause this repression, since ABA has been shown to negatively impact the expression of genes involved in N metabolism (Ristova *et al.*, 2016).

Importantly, knocking out genes involved in the N uptake/assimilation pathway leads to improved drought responses. For example, mutations in the N transceptor NRT1.1/NPF6.3 allows plants to withstand W stress; they exhibit an enhanced drought tolerance phenotype, as compared with wild-type plants (Guo *et al.*, 2003). The *NRT1.1/NPF6.3* gene encodes a dual-affinity nitrate transporter that contributes to both low- and high-affinity uptake in Arabidopsis roots (Tsay *et al.*, 1993). NRT1.1/NPF6.3 is also a key component of N signaling because it functions as a nitrate sensor of a wide range of concentrations in roots (Ho *et al.*, 2009). NRT1.1/NPF6.3 was found to be expressed in guard cells of leaves, where it plays a role in nitrate accumulation during stomatal aperture through nitrate-induced membrane depolarization. In the *nrt1.1/npf6.3* mutant background, stomata close and transpiration rates decline, thus allowing plants to conserve W (Guo *et al.*, 2003). The transcription factor (TF) gene *NIN-LIKE PROTEIN 7* (*NLP7*), which regulates NRT1.1/NPF6.3 expression as well as other genes involved in N metabolism, also impacts drought responses (Castaings *et al.*, 2009). Like *nrt1.1* mutants, *nlp7* mutants transpire less and survive longer under drought (Castaings *et al.*, 2009). Similar to NRT1.1/NPF6.3, NLP7 is expressed in guard cells. Probably, NLP7 controls stomatal aperture in response to N through regulating NRT1.1/NPF6.3 expression (Castaings *et al.*, 2009; Marchive *et al.*, 2013) (Fig. 1B).

Like *NLP7* and *NRT1.1/NPF6.3*, mutations in *NIA1* and *NIA2*, genes encoding nitrate reductase (NR), the enzyme responsible for reducing nitrate to nitrite, also produce a drought resistant phenotype (Fig. 1B) (Lozano-Juste and León, 2010). Compared with the wild type, the *nia1*/*nia2* mutants exhibit a smaller shoot biomass and a lower rate of N assimilation. Being smaller plants, their physiology allows them to survive longer under drought (Lozano-Juste and León, 2010). However, their improved drought tolerance may not just be due to their smaller size; it may also be due an to enhanced sensitivity to ABA. This is because besides it role in reducing nitrate, NR activity also contributes to the generation of nitric oxide (NO), which is a negative regulator of ABA signaling (Lozano-Juste and León, 2010). Thus, *nia1/nia2* mutants produce less NO, leading to increased ABA activity, stomatal closure, and enhanced drought tolerance (Castillo *et al.*, 2015; Chen *et al.*, 2016).

Collectively, these insights indicate that genes involved in N uptake/metabolism and N signaling also play a role in drought responses. At least part of this crosstalk can be explained by N signaling genes regulating ABA and drought responses at a transcriptional level, as described below.

## N and W signals converge at the transcriptome level

Over the past decade, transcriptomic approaches have been employed to investigate how plants signal changes in N or W availability at the molecular level. These studies have revealed that thousands of genes are differentially expressed in response to either changes in N dose or W status, highlighting that the gene regulatory networks that govern responses to N and W are highly complex (Vidal and Gutierrez, 2008; Wilkins *et al.*, 2010; Sharma *et al.*, 2018; Swift *et al.*, 2019).

**Fig. 2.**
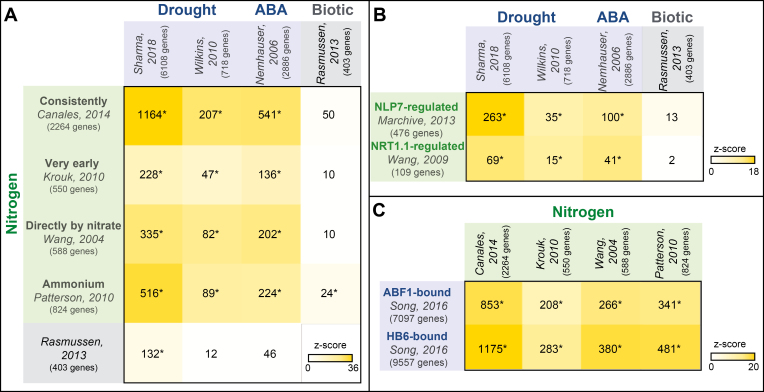
N and W are convergent signals at the transcriptomic level. Four published data sets of N-regulated genes in Arabidopsis were collected. These data represent (i) genes consistently regulated by N across independent studies (Canales *et al.*, 2014); (ii) very early N-regulated genes identified in a time-series experiment (Krouk *et al.*, 2010); (iii) genes directly regulated by nitrate (Wang *et al.*, 2004); and (iv) genes regulated by ammonium (Patterson *et al.*, 2010). N-regulated genes were compared with two independent data sets of drought-regulated genes in Arabidopsis (Wilkins *et al.*, 2010; Sharma *et al.*, 2018); one data set of ABA-responsive genes (Nemhauser *et al.*, 2006); and one data set of genes responsive to biotic stress (included here as a negative control) (Rasmussen *et al.*, 2013). Intersect results of N-regulated genes with drought, ABA, and biotic response gene sets are presented as a heatmap, where both the number of genes and the significance of the overlap are indicated (*Monte-carlo *P*<0.001) with the *z*-score value for each comparison). (A) Genes regulated by N are highly enriched in drought and ABA-responsive genes. (B) N-response genes regulated by the master TF of the N-response NLP7 (Marchive *et al.*, 2013) or the nitrate transceptor NRT1.1/NPF6.3 (Wang *et al.*, 2009) are highly enriched in drought- and ABA-responsive genes (Nemhauser *et al.*, 2006; Wilkins *et al.*, 2010; Sharma *et al.*, 2018). (C) ABA-responsive genes that are bound by the TFs ABF1 or HB6 (Song *et al.*, 2016) are highly enriched in N-regulated genes (Wang *et al.*, 2004; Krouk *et al.*, 2010; Patterson *et al.*, 2010; Canales *et al.*, 2014).

As described above, the availability of N and W has combinatorial effects on plant physiology. Thus, it is possible that N and W also have combinatorial effects on transcriptomic responses. To investigate this hypothesis, taking advantage of existing RNA sequencing (RNA-seq) and microarray data sets detailing N-responsive or W-responsive genes in Arabidopsis, a simple meta-analysis was performed as part of this review (Fig. 2). To evaluate whether gene expression patterns responsive to N were impacted by W (and vice versa), Arabidopsis genes reported to be responsive to either N, drought, or the drought signaling hormone ABA were overlapped (Fig. 2B).

The results of this meta-analysis indicated that Arabidopsis relies on the same set of genes to signal both N and W (Fig. 2A). Specifically, 51% of the 2264 genes reported to be differentially expressed in response to N are also drought responsive (Fig 2A)—a significantly higher overlap than expected by chance (Canales *et al.*, 2014; Wilkins *et al.*, 2010; Sharma *et al.*, 2018). This suggests that many genes responsive to N are also drought responsive (Fig. 2A). This effect held true regardless of whether the N source was nitrate (Wang *et al.*, 2004) or ammonia (Patterson *et al.*, 2010) (Fig. 2A). Furthermore, a significant overlap was found between drought-responsive genes and genes differentially expressed within minutes of N treatment (Krouk *et al.*, 2010), suggesting that genes immediately downstream of N sensing are also drought responsive (Fig. 2A). Additionally, the meta-analysis showed that genes immediately downstream of W sensing intersect with N-responsive genes. Specifically, the meta-analysis showed that N-regulated genes are highly enriched in ABA-responsive genes (Nemhauser *et al.*, 2006). This finding agrees with the combinatorial effect that N and ABA treatments are reported to have on gene expression in Arabidopsis roots (Krouk *et al.*, 2010; Ristova *et al.*, 2016).

Additional evidence was inspected to support this finding. Specifically, it was assessed whether ABA-responsive TFs regulate N-responsive genes (Fig. 2C). The ABF1 and HB6 TFs were selected to test this hypothesis, based on their reported function in drought tolerance and ABA signaling (Choi *et al.*, 2000; Himmelbach *et al.*, 2002; Lechner *et al.*, 2011; Yoshida *et al.*, 2015; Song *et al.*, 2016). Since a significant overlap was found of ABF1-bound and HB6-bound genes with N-regulated genes, this suggests that ABA signaling TFs have a role mediating N-responsive gene expression patterns (Fig. 2C). This finding agrees with a previous study showing a significant enrichment of the *cis*-regulatory elements recognized by ABA-responsive TFs in the promoter of N-regulated genes (Nero *et al.*, 2009).

Finally, an additional analysis was performed to support the conclusion that perturbing N uptake and signaling genes impacts drought responses. As discussed above, mutations in the TF NLP7 and N transceptor NRT1.1/NPF6.3 not only decrease the ability of Arabidopsis to acquire N from the environment, but also improve their survival under drought stress (Guo *et al.*, 2003; Castaings *et al.*, 2009; Wang *et al.*, 2009; Marchive *et al.*, 2013; Bouguyon *et al.*, 2015). N-regulated genes whose expression is mediated by NLP7 or NRT1.1/NPF6.3 (Wang *et al.*, 2009; Marchive *et al.*, 2013) were tested for enrichment in drought- or ABA-regulated gene sets (Wang *et al.*, 2009; Marchive *et al.*, 2013). As expected, a significant proportion of NLP7-dependent or NRT1.1/NPF6.3-dependent genes are differentially expressed in response to drought or ABA treatment (Fig. 2B).

Collectively, these published studies and new analyses conducted herein suggest that perturbing key N signaling genes leads to changes in drought-responsive gene expression programs. This may explain why drought tolerance is improved in these N signaling mutants, as discussed above. These new genome-wide analyses highlight the importance of investigating the signaling crosstalk between N and W signals.

## N and W have non-linear effects on gene expression and crop phenotypes

Since there is evidence that transcriptional networks that signal N and W status overlap (Fig. 2), could such genes be responsible for the combinatorial effects that N and W have on plant physiology? This question was recently addressed in a single, integrated study in rice, which directly investigated the combinatorial effects that N and W have at both the transcriptional and physiological level (Swift *et al.*, 2019). Testing how N and W combine to affect plant biology is challenging, as N is dissolved in W. Thus, while changing the amount of W available does not impact the amount of N present, it will change the concentration of N (Swift *et al.*, 2019). Indeed, this raises the important question about how plants respond to N dose in the context of a changing W environment—as N moles, or as N concentration (i.e. the amount of N dissolved W, or ‘N/W’).

Through a factorial matrix experimental design that systematically varied both N and W (Fig. 1A.1), the rice study was able to uncouple the effects of N amount (moles) versus N concentration (N/W) (Swift *et al.*, 2019). By these means, it was revealed that both N amount and N concentration had distinct dose-responsive effects on plant transcriptomic responses (Fig. 1A.2), where canonical N-responsive genes were found in both of these types of gene expression responses (Swift *et al.*, 2019). Additionally, the authors found that the amount of N and W had a synergistic effect on gene expression responses (modeled as ‘N×W’). In other words, a subset of genes were differentially expressed only when N and W were both present in sufficiently high amounts.

Indeed, rice genes whose expression responses were explained by the presence of N and W in combination—as N concentration (N/W), or synergistically (N×W)—outnumbered those genes whose expression depended on either N or W alone (Fig. 1A.2). This finding agrees with the meta-analysis of N and W response genes in Arabidopsis presented in Fig. 2, which suggests that many genes are transcriptionally responsive to both N and W status. Importantly, in the rice study, it was shown that genes whose expression pattern depended on both N and W status were better predictors of rice traits under field conditions—such as grain yield and crop biomass—compared with genes that responded only to N or only to W (Swift *et al.*, 2019). Thus, the study of Swift *et al.* demonstrates that the combinatorial effects that N and W have on rice phenotype, as described above, are possibly directed through integrating N and W status at the molecular level.

## Future challenges: adapting crops to dry, low N soils

N-based fertilizers are not only expensive, their use negatively impacts the biosphere (Robertson and Vitousek, 2009; Sutton *et al.*, 2011). At the same time, climate change is predicted to lead to drier soils around the globe (Ulrich *et al.*, 2014). Thus, developing crops that require both less N and less W can help adapt agriculture to these changes. To meet this challenge, what specific plant traits might be targeted? And what research strategies could help identify useful loci?

In terms of the traits to target, focusing on root traits is beneficial because of the large effects N and W have on root development. Since W carries N down deeper into the soil as it dries, breeding plants with deeper root systems may adapt plants to low N, low W conditions. In some instances, increasing root depth has been shown to be beneficial for adapting rice to low W conditions (Arai-Sanoh *et al.*, 2014). Similarly, the ability to elongate roots into deeper soil layers has been linked to improved N acquisition (Guo and York, 2019). Studying root system architecture at a high resolution is laborious and low throughput. For this reason, the use of 3-D simulated root systems, such as OpenSimRoot (Postma *et al.*, 2017), which can model root architecture responses to soil types across a variety of conditions, are becoming a valuable resource. Simulated root systems have been used to study the effect of the availability of N and other nutrients on root growth (Postma *et al.*, 2014). Since this system calculates nutrient and W uptake as the roots grow and receive photosynthate from the shoot in a virtual 3-D soil environment, it may assist in quickly parsing out how different combinations of N and W impact plant performance.

In contrast to root systems, which typically expand under low N, low W conditions, plants will reduce their growth of aerial tissue. Smaller shoot systems transpire less, adapting plants to an environment with less W (Shangguan *et al.*, 2000). Similarly, when N is limited, leaf tissue will senesce, reducing aerial tissue size and boosting N remobilization (Park *et al.*, 2018). However, evidence suggests that plants undergo this phenotypic change even under mild stress conditions, where N and W remain available for growth (Shi *et al.*, 2014). While such bet-hedging strategies ensure plant survival in natural settings, when stress occurs in agricultural settings this mechanism unnecessarily limits crop growth and yield (Cabrera-Bosquet *et al.*, 2007; Skirycz and Inzé, 2010). Accordingly, transgenic plants displaying enhanced drought resistance phenotypes usually exhibit lower growth (Tardieu, 2012), highlighting the need for strategies to uncouple drought resistance from growth. In this regard, understanding the impact of N signaling versus N growth on drought resistance could be a key step for engineering plants with improved growth in low N and low W environments.

Another approach to adapt shoot systems to low N, low W soils could be to reduce stomatal density. A higher number of stomata per unit leaf area leads to higher transpiration rates, as well as a higher sensitivity to drought (Henry *et al.*, 2019). It is thought that for this reason, drought-adapted species hold fewer stomata (Henry *et al.*, 2019). In line with this, plants with less than half of their normal density of stomata have reduced levels of transpiration, and are more drought tolerant (Yu *et al.*, 2008). Importantly, these benefits were observed without a change in shoot N concentration (Hepworth *et al.*, 2015). One proposed way to engineer this trait is through overexpressing EPF1, a signaling peptide which controls the frequency of stomata in developing leaves (Simmons and Bergmann, 2016). EPF1 overexpression lines in barley have lower stomatal density and enhanced drought tolerance (Hughes *et al.*, 2017). Despite substantial reductions in leaf gas exchange, barley plants with reduced stomatal density show no reductions in grain yield (Hughes *et al.*, 2017).

Another strategy for improving growth on low N and drought is by tapping an important source of crop genetic diversity which resides in their wild varieties. Alleles that could enhance growth under low N, low W environments may be absent from many modern cultivars, owing to their loss during domestication or subsequent germplasm improvement (Díaz *et al.*, 2019). To address this, many breeding programs now exploit wild varieties of domesticated crops (Kumar *et al*., 2008, 2014). Additionally, novel loci conferring tolerance to low N and low W environments may be found in gene pools of native plants endemic to arid lands (Uga *et al.*, 2011). Plants native to deserts must cope with low precipitation and poor nutritional soils—as well as additional factors such as high temperatures, salinity, and high light intensity. An exemplary case is the Atacama desert in northern Chile. Some parts of this desert have had no recorded rainfall in the last 30 years. Furthermore, it has an extremely low soil N concentration—almost 20 times less than fertilized soils (Díaz *et al.*, 2016). Investigating the means by which plant species have adapted to this extreme environment may identify new molecular mechanisms that are useful for adapting crops to low N, low W environments. Along these lines, genomic studies have begun to be conducted on individual desert species (Mickelbart *et al.*, 2015). Investigating plant species from diverse phylogenetic origins and their adaptation to combined stress will strengthen the quest for finding signature gene functions underlying plant survival in dry, nutrient-poor marginal environments.

## Concluding remarks

Here, we have discussed the combinatorial effects that N and W can have on plant physiology, and shed light on the possible molecular mechanisms and transcriptome signaling interactions that underlie these responses.

Root architecture shows high developmental plasticity to combined N and W availability. At least part of these adaptations may be the result of co-ordination between transcriptional programs in response to N and W signals. Known signaling components of N and ABA signaling pathways may be involved in this coordination. The NLP7 TF and NRT1.1 nitrate transceptor mediate the expression of N-responsive genes, and—as proposed herein through genome-wide analysis—the expression of drought-responsive genes. Similarly, our analysis showed that TFs in the ABA pathway mediate drought responses as well as the expression of N-responsive genes. The effect of perturbing signaling components of one pathway and evaluating the impact on the other at a genome-scale level—in a single experimental design—remains to be determined. Such an approach would provide further insight into how plants coordinate transcriptional programs to adapt organ responses to changes in both N and W availability. We speculate that as our understanding of signaling pathways in different crops grows, species will differ in how they integrate N and W signals at the molecular level. These differences may in turn explain why some crop varieties vary in their phenotypic responses to N and W combinations (Swift *et al.*, 2019).

As discussed in this review, there is strong evidence that the molecular signaling pathways that respond to N are contingent on W, and vice versa. On one hand, this additional layer of complexity means that untangling how plants signal different environment factors at the molecular level may prove extremely challenging. On the other, delineating which genes integrate these essential environmental signals may prove a promising means to adapt crops to multiple environmental stresses. This will be increasingly important to maintain or increase crop productivity in the face of climate change.
